# Migration of nonylphenol and plasticizers from polyvinyl chloride stretch film into food simulants, rapeseed oil, and foods

**DOI:** 10.1002/fsn3.404

**Published:** 2016-07-05

**Authors:** Yoko Kawamura, Yuko Ogawa, Motoh Mutsuga

**Affiliations:** ^1^ Division of Food AdditivesNational Institute of Health SciencesTokyoJapan; ^2^ Present address: Faculty of Pharmaceutical SciencesTeikyo Heisei UniversityTokyoJapan

**Keywords:** Diacetyllauroyl glycerol, diisononyl adipate, di‐*n*‐alkyl adipate, nonylphenol, polyvinyl chloride stretch film

## Abstract

Nonylphenol (NP) has been suspected as an endocrine‐disrupting chemical. Japanese polyvinyl chloride (PVC) stretch films contained 0.5–3.3 mg/g of NP and 100–400 mg/g of plasticizers such as diisononyl adipate (DINA), di‐*n*‐alkyl adipate (DAA), and diacetyllauroyl glycerol (DALG). Migration of NP and plasticizers from PVC stretch films into food simulants (water; 4% acetic acid; 20%, 50%, and 95% ethanol; and heptane), rapeseed oil, and foods was investigated. Plasticizers migrated only in small amounts into aqueous simulants and foods, although they migrated at much higher levels into 50% and 95% ethanol, heptane, rapeseed oil, and fatty foods, whereas NP more easily migrated into aqueous simulants and foods. At 5°C for 24 hr, migration of NP into vegetable and fruit was 2.9%–6.4% of their contents, and that of DINA and DAA was 0.1%–0.3%. The migration ratios of NP into aqueous foods were much higher than those of DINA and DAA. The migration ratio of NP into fatty foods, such as minced tuna and pork, was 33% and 24%, which was almost similar to that of DINA and DAA. The estimated daily intakes of NP and DINA for Japanese individuals of those days were 35 and 1,050 μg, respectively, and should not be associated with any safety concerns.

## 1 Introduction

**Figure 1:**
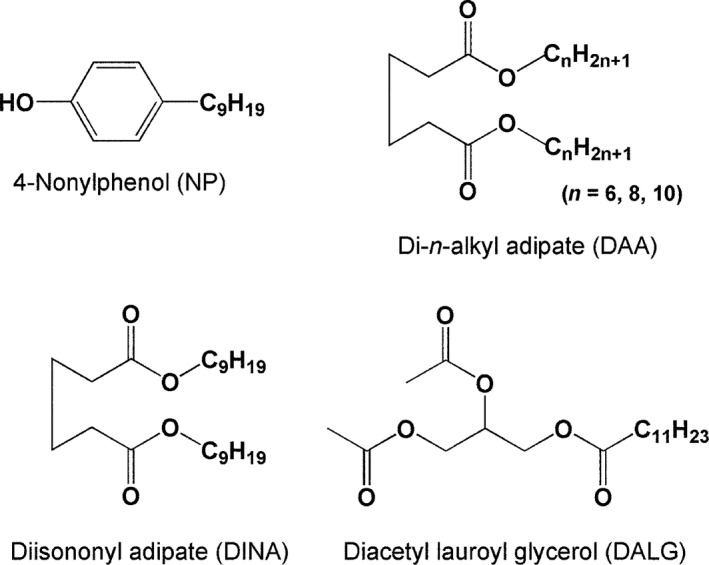
Structures of nonylphenol, diisononyl adipate, di‐*n*‐alkyl adipate, and diacetyl lauroyl glycerol

Nonylphenol (NP) is a complex mixture predominantly containing 4‐nonylphenol (>90%) with varied alkyl chain branching (Fig. 1). NP is widely used in many industrial applications including nonylphenol ethoxylates for nonionic surfactants and tris(nonylphenyl) phosphite for antioxidants. These chemicals breakdown into NP, which then contaminates the environment and food.

NP has been detected in many plastics intended to come into contact with food, including polycarbonate tableware and baby bottles, polystyrene disposable cups and food cases, polyvinyl chloride (PVC) stretch films and gloves (Fernandes, et al., 2008; Kao 2012; Kawamura et al., 1999; Kawamura et al., 2000a,b; UKFSA 2010). Among them, the PVC stretch films contain NP most frequently and the biggest amount (Inoue et al., 2001, Kawamura et al., 1999, Kawamura et al., 2000a, Votavová et al., 2009). NP in PVC films is a nonintentionally added substance (NIAS), which is a degradation product of the antioxidant tris(nonylphenyl) phosphite (Kawamura et al., 2000aa; McNeal et al., 1999; Mottier et al., 2014).

NP has been suspected as an endocrine‐disrupting chemical, which induced cell proliferation and the progesterone receptor in MCF_7_ breast tumor cells and mitotic activity in rat endometrium (Soto et al., 1991). Moreover, it stimulated vitellogenin gene expression in trout hepatocytes (White et al., 1994) and inhibited testicular growth (Jobling et al., 1996). On the basis of these studies, NP has become a social concern as a suspected endocrine‐disrupting chemical, and Japanese consumers have refused products containing NP since the end of 1990s. Therefore, the Japan Vinyl Goods Manufacturers Association changed the composition of PVC stretch films in 2002; since then, NP has not been detected in Japanese stretch films. However, PVC films containing NP were popular in Japan during the 20 years from the early 1980s to 2002 exposing the Japanese population to high NP amounts.

Japanese PVC stretch films also contained 100–400 mg/g of plasticizers, mainly comprising diisononyl adipate (DINA) and di‐*n*‐alkyl adipate (C = 6, 8, and 10) (DAA), di‐*n*‐octyl adipate, heptylnonyl adipate, and/or diacetyllauroyl glycerol (DALG) (Kawamura et al., 1999) (Fig. 1).

Studies have reported the migration of NP and/or plasticizers from PVC stretch film into food simulants and food (Inoue et al., 2001, Saito et al., 2002, Funayama et al., 2002, Votavová et al., 2009). In this study, the migration of NP and plasticizers, such as DINA, DAA, and DALG, from PVC stretch films into food simulants, rapeseed oil, and several foods were circumstantially investigated under various conditions and their intakes were estimated. This study was conducted from 1999 to 2001.

## 2 Materials and Methods

### 2.1 Samples

Two PVC stretch films were purchased from the Japanese market in 1999. Film 1 contained 2.1 mg/g (0.28 mg/dm^2^) of NP, 105 mg/g (14.1 mg/dm^2^) of DINA, and 16.8 mg/g (2.3 mg/dm^2^) of DAA, and film 2 contained 1.5 mg/g (0.21 mg/dm^2^) of NP, 130 mg/g (18.2 mg/dm^2^) of DINA, and 16.0 mg/g (2.2 mg/dm^2^) of DALG. They were typical components of PVC stretch films in the 1990s in Japan.

### 2.2 Chemicals

NP (>95% of 4‐nonylphenol, mixture of branched chain isomers, CAS 84852‐15‐3), DINA (>98%, mixture of branched chain isomers, CAS 33703‐08‐1), DAA (>95%, mixture of adipic acid di *n*‐hexyl/octyl/decyl esters), and DALG (mixture of analogs, CAS 30899‐62‐8) were purchased from Tokyo Kasei Kogyo (Tokyo, Japan) or Sigma‐Aldrich Co. LLC. (St. Louis, MO, USA). Each compound was dissolved in hexane, and diluted with hexane to obtain a calibration curve or with acetone for addition in recovery tests. Anhydrous sodium sulfate (pesticide analysis grade) that was purchased from Wako Pure Chemicals (Osaka, Japan) was heated at 180°C overnight before use. A solid‐phase cartridge containing styrene‐divinylbenzene was obtained from GL Science (Tokyo, Japan) and was conditioned before use with 5 ml of acetonitrile followed by 5 ml of water.

A Florisil column was prepared as follows. Florisil PR^®^ (60–100 mesh, Floridin Co., NY, USA) was heated at 180°C overnight and cooled in a desiccator, and 6% (w/w) ultrapure water purified with MILLI‐Q SP (Millipore, Billerica, USA) was added and mixed well. A glass column (i.d., 1 cm) was packed with 2.0 g of Florisil containing water precisely, followed by ca. 1 g of anhydrous sodium sulfate on a glass wool plug. The column was then conditioned with 10 ml of hexane.

### 2.3 Rapeseed oil and foods

Rapeseed oil was vented in polyethylene bottles for cooking use by Nissin Oilio Group Ltd. (Tokyo, Japan). Vegetables and fruits (Japanese radish, pumpkin, pineapple, and melon), cooked food (cooked radish with fried tofu, meat sauce, hamburger steak, croquette, and potato salad), and fresh meat and fish (minced chicken white meat, minced pork, and minced tuna) were obtained at local markets.

### 2.4 Apparatus

A migration cell with 1 dm^2^‐migration area was obtained from Maeda Seisakusyo (Tokyo, Japan). The centrifugal condenser CVE‐100, Tokyo Rikakiki Co. (Tokyo, Japan) and the GC/MS system comprising GC 6890 and MS 5973 (Agilent Technologies, USA) were used.

### 2.5 GC/MS conditions

The GC/MS system was equipped with a fused‐silica column DB‐1 (0.25 mm i.d. × 5 m, 0.1 μm film thickness, Agilent Technologies). The oven temperature was maintained at 50°C and then ramped at 20°C/min to 300°C. The injection port and interface were kept at 250°C and 280°C, respectively. The carrier gas was 3 ml/min helium (1 psi constant). The injection mode was splitless with a 1 min sampling time and the injection volume was 1 μl. Mass spectrometric conditions were as follows: electron ionization mode, ionization voltage 70 eV, and detection voltage 2.0 kV. The quantification ions were NP at m/z 135 and 149, DINA at m/z 255, DAA at m/z 129, and DALG at m/z 159. The confirmation ions were NP at m/z 107 and 121, DINA at m/z 129, DAA at m/z 213, and DALG at m/z 183.

### 2.6 Migration test into food simulants using a migration cell

Stretch film (film 1 or 2) was fixed in the migration cell, in which 100 ml of the simulant was poured and maintained at the test temperature. Test conditions were 60°C or 95°C for 30 min with water; 60°C for 30 min with 4% acetic acid, 20%, 50%, and 95% ethanol, and rapeseed oil; or 25°C for 60 min with heptane. These are the same conditions that are specified by the Japanese regulations, except for the 50% and 95% ethanol, and raapeseed oil. The migration solutions were prepared for the test solutions as follows and were determined using GC/MS.

- With 95% ethanol and heptane: These were diluted or evaporated with a rotary evaporator under 40°C to appropriate concentrations for GC/MS analysis.
- With water and 4% acetic acid: These were poured in a conditioned solid‐phase cartridge under vacuum, and the eluents were discarded. NP and plasticizers were eluted with 5 ml of acetonitrile and evaporated at room temperature under nitrogen to 1 ml.
- With 20% and 50% ethanol: These were diluted to less than 10% of ethanol concentration with water and were prepared in the same way as with water and 4% acetic acid.
- With rapeseed oil: It (2 g) was transferred to a separating funnel containing 20 ml of hexane. Acetonitrile saturated with hexane (30 ml) was added and shaken, and the acetonitrile layer was separated. This procedure was repeated thrice. The acetonitrile layers were transferred to another separating funnel containing 250 ml of 10% NaCl solution, followed by two extractions with 100 ml of hexane each. The hexane extracts were dehydrated with anhydrous sodium sulfate and were evaporated with a rotary evaporator under 40°C to approximately 5 ml. They were poured in a Florisil column and eluted with 50 ml of hexane. The eluents were discarded. NP and plasticizers were eluted with 50 ml of 1% acetonitrile in hexane. The eluates were evaporated at room temperature under nitrogen to 1 ml.

### 2.7 Migration test into rapeseed oil in Petri dish

The stretch film (film 1, 18 cm^2^) covered 16.5 g (18 ml) of rapeseed oil in a Petri dish and was maintained at the test conditions of at −20 and 5°C for 30 min and 1, 14, and 28 days; at 25°C for 30 min and 2, 4, and 16 hr; at 60°C for 10, 30, and 60 min and 2 hr; and at 95°C for 5, 10, 30, and 60 min. After migration, rapeseed oil was prepared described above and was analyzed using GC/MS.

### 2.8 Migration test into foods

The stretch film (film 1, 18 cm^2^) covered 20 g of food in a Petri dish at 5°C for 24 hr. Cooked hot food (20 g) was immediately covered with the film and stored at room temperature for 30 min. The test solutions were prepared as follows and analyzed using GC/MS. Analytical data were corrected for their recovaries.

- Non‐fatty food: After the contact time, the food was transferred to a blender cup containing 50 ml of acetone, homogenized and centrifuged at 1000 g for 10 min. The acetone layer was separated, and the residue was mixed again with 50 ml of acetone and centrifuged. The acetone layers were combined and evaporated with a rotary evaporator under 40°C to 30 ml. This concentrate was transferred to a separating funnel containing 250 ml of 10% NaCl solution. It was then extracted with hexane and purified using a Florisil column, following the rapeseed oil procedures in this step.
- Fatty food: After the contact time, the food was transferred to a blender cup containing 50 ml of hexane, homogenized and centrifuged at 1000 g for 10 min. The hexane layer was separated, and the residual homogenate was mixed again with 50 ml of hexane and then centrifuged. The hexane layers were combined, dehydrated with anhydrous sodium sulfate, filtered and expanded up to 200 ml with hexane. A 20‐ml solution was transferred to a separating funnel, and 30 ml of acetonitrile saturated with hexane was added. Subsequently, the test solution was prepared according to the rapeseed oil procedure from this step.

### 2.9 Quality control and quality assurance

Every migration test was performed at least thrice. To determine the quality of the method, each extraction and clean up procedure of rapeseed oil and foods was accompanied by a blank and recovery test. The recovery test was performed for rapeseed oil that was spiked with NP, DINA, DAA, and DALG standards, and all foods that were spiked with NP, DINA, and DAA standards. The recoveries and SD are presented in Table 1. The mean recoveries ranged from 39.9% to 96.8% with an SD under 9.7%. Most recoveries of NP, DAA, and DALG were more than 60% and were considered satisfactory, whereas those of DINA were sometimes less than 50%. Therefore, the analytical data of NP, DINA, DAA, and DALG in rapeseed oil and foods were corrected by their rates of recoveries. The limits of quantification (LOQ) were 0.5 μg/dm^2^ for NP, DAA, and DALG, and 1.0 μg/dm^2^ for DINA in water and solvents; 1.0 μg/dm^2^ for NP, DAA, and DALG, and 2.0 μg/dm^2^ for DINA in non‐fatty foods; and 2.5 μg/dm^2^ for NP, DAA, and DALG, and 5.0 μg/dm^2^ for DINA in rapeseed oil and fatty foods. LOQ was set to more than 10 times the noise levels.

**Table 1:** Recoveries of NP, DINA, DAA, and DALG spiked in rapeseed oil and various foods

Foods	Spiked amount	Recovery (%)			
	(μg)	NP	DINA	DAA	DALG
Rapeseed oil	10	74.6 ± 5.3	51.3 ± 3.8	61.5 ± 4.7	71.3 ± 6.9
Rapeseed oil	1000	86.8 ± 2.3	49.8 ± 4.4	74.6 ± 3.4	89.5 ± 2.6
Japanese radish	10	88.4 ± 6.7	56.9 ± 9.7	73.8 ± 7.3	—
Pumpkin	10	68.7 ± 6.3	54.5 ± 2.0	64.5 ± 4.9	—
Pineapple	10	47.4 ± 2.4	43.9 ± 3.3	44.0 ± 0.9	—
Melon	10	53.2 ± 5.0	41.3 ± 2.7	39.9 ± 3.2	—
Cooked radish with fried tofu	100	79.4 ± 2.1	47.6 ± 0.8	64.9 ± 1.9	—
Meat sauce	100	92.9 ± 4.8	70.1 ± 3.2	79.7 ± 1.3	—
Hamburger	100	85.7 ± 2.1	50.5 ± 2.5	75.9 ± 2.9	—
Potato salad	100	96.8 ± 1.9	60.4 ± 4.4	70.6 ± 1.8	—
Croquette	100	77.8 ± 0.2	44.1 ± 2.2	67.9 ± 2.0	—
Minced chicken white meat	100	77.7 ± 0.1	50.0 ± 0.4	71.8 ± 1.8	—
Minced tuna	100	90.9 ± 4.0	48.8 ± 2.6	69.8 ± 5.0	—
Minced pork	100	89.1 ± 1.9	52.7 ± 0.2	67.9 ± 1.5	—

Each value is the mean ± SD of three or more determinations.

—: not tested.

## 3 Results and Discussion

### 3.1 Quantifications of NP, DINA, DAA, and DALG by GC/MS

**Figure 2:**
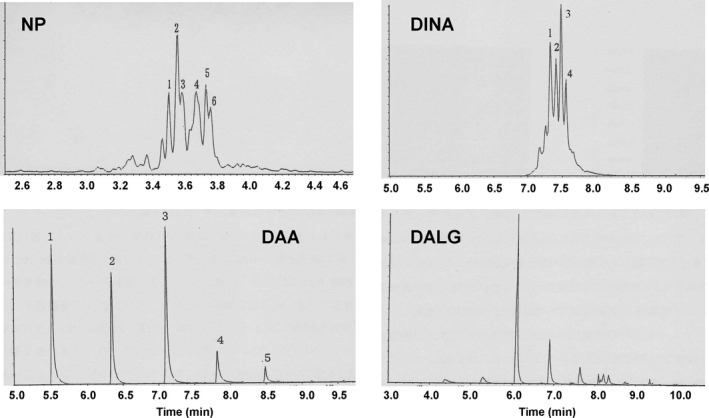
GC/MS ion chromatograms of NP, DINA, DAA, and DALG standards. NP, nonylphenol; DINA, diisononyl adpate; DAA, di‐n‐alkyl adipate; DALG, diacetyllauroyl glycerol

NP, DINA, DAA, and DALG are mixtures of isomers or analogs; each chemical thus showed many peaks of particular shape on GC/MS ion chromatograms (Fig. 2). Moreover, some peaks were affected by other chemicals. Therefore, they were quantified using their ion chromatograms. NP was quantified by the total peaks of m/z 135 and 149 between 3.4 and 3.8 min, DINA was quantified by the total peaks of m/z 255 between 7.0 and 8.2 min, DAA was quantified by the two peaks of m/z 129 at 5.5 and 6.3 min, and DALG was quantified by the highest peak of m/z 159 at 6.1 min.

### 3.2 Migrations of NP, DINA, DAA, and DALG from PVC stretch films into food simulants

Migrations of NP, DINA, DAA, and DALG from PVC stretch films into food simulants were tested using a migration cell (Table 2). Rapeseed oil was used instead of olive oil because di‐2‐ethylhexyl phthalate (DEHP) was detected in several brands of the test reagent‐grade olive oil, although no DEHP, NP, DINA, DAA, or DALG was detected in rapeseed oil that was packed in a polyethylene bottle for cooking. The percentage of migration ratio was calculated using the following formula:

$$\text{Migration ratio}\;\left(\%\right)\;=\;\frac{\text{migration level}(\mu g/{\text{dm}}^{2})}{\text{material content}(\mu g/{\text{dm}}^{2})}\;\times \;100$$

**Table 2:** Migration of NP, DINA, DAA, and DALG from polyvinyl chloride stretch films into food simulants

Film No.	Simulant	Temp (°C)	Time (min)	Migration level (μg/dm^2^) (Migration ratio, %)			
				NP	DINA	DAA	DALG
1	Water	60	30	2.1 ± 0.0 (0.8)	1.1 ± 0.0 (0.01)	0.5 ± 0.0 (0.02)	—
	Water	95	30	3.4 ± 0.1 (1.2)	1.2 ± 0.0 (0.01)	0.6 ± 0.0 (0.03)	—
	4% Acetic acid	60	30	5.3 ± 0.1 (1.9)	4.3 ± 0.2 (0.03)	0.5 ± 0.0 (0.02)	—
	20% Ethanol	60	30	8.0 ± 0.1 (2.9)	5.9 ± 0.1 (0.04)	0.6 ± 0.0 (0.03)	—
	50% Ethanol	60	30	237 ± 13 (85)	2160 ± 70 (16)	900 ± 28 (39)	—
	95% Ethanol	60	30	254 ± 9 (91)	7810 ± 120 (58)	1510 ± 50 (66)	—
	Heptane	25	60	267 ± 10 (95)	12900 ± 500 (95)	2160 ± 90 (94)	—
	Rapeseed oil	60	30	199 ± 15 (71)	9650 ± 320 (68)	1540 ± 70 (67)	—
2	Water	60	30	1.3 ± 0.0 (0.6)	1.5 ± 0.0 (0.01)	—	1.0 ± 0.0 (0.05)
	Water	95	30	3.3 ± 0.1 (1.6)	1.5 ± 0.1 (0.01)	—	1.7 ± 0.1 (0.08)
	4% Acetic acid	60	30	3.4 ± 0.1 (1.6)	5.0 ± 0.2 (0.03)	—	2.3 ± 0.1 (0.10)
	20% Ethanol	60	30	7.6 ± 0.3 (3.6)	11.4 ± 0.6 (0.06)	—	6.8 ± 0.4 (0.29)
	50% Ethanol	60	30	180 ± 8 (86)	2700 ± 140 (15)	—	1200 ± 50 (55)
	95% Ethanol	60	30	198 ± 14 (94)	11800 ± 300 (65)	—	1670 ± 70 (76)
	Heptane	25	60	205 ± 9 (98)	16100 ± 700 (88)	—	2090 ± 100 (95)
	Rapeseed oil	60	30	157 ± 11 (75)	12100 ± 500 (67)	—	1390 ± 80 (63)

Each value is the mean ± SD of three or more determinations.

( ) is migration ratio (%) = migration level (μg/dm^2^)/material content (μg/dm^2^) × 100.

—: not contained.

DINA and DAA migrated only in low amounts into aqueous simulants such as water, 4% acetic acid, and 20% ethanol, and even in 95°C water. The migration levels were under 11.4 μg/dm^2^, and the migration ratios were under 0.06%. DALG migrated into aqueous simulants in rather higher ratios than DINA and DAA. These plasticizers exhibited in extremely high migration into lipid‐soluble simulants such as 50% and 95% ethanol and heptane, with migration increasing in this order; their migration levels ranged from 900 to 16,100 μg/dm^2^, and their migration ratios were 14%–52%. The plasticizers migrated at levels from 672 to 6,030 μg/dm^2^ into the rapeseed oil, and their migration ratios were 16%–95%. Their levels in rapeseed oil were from 1,390 to 12,100 μg/dm^2^ and their migration ratios were between 63% and 68%.

The migration levels of NP into aqueous simulants were 1.3–8.0 μg/dm^2^, and their migration ratios were 0.6%–3.9%, which were almost 10–100 times higher than these of plasticizers under the same condition. The migration levels of NP into lipid‐soluble simulants were 180–267 μg/dm^2^, and their ratios were greater than 85%. Most NP also migrated into lipid‐soluble simulants. The migration levels in rapeseed oil were 199 and 157 μg/dm^2^ (71% and 75%).

### 3.3 Migrations of NP, DINA, and DAA from PVC stretch films into rapeseed oil

The migration levels and ratios of NP and plasticizers from PVC stretch films into the rapeseed oil at −20 to 95°C for various periods are shown in Table 3.

**Table 3:** Migrations of NP, DINA, and DAA from polyvinyl chloride stretch film into rapeseed oil under various conditions

Temp(°C)	Time	Migration level (μg/dm^2^) (Migration ratio, %)		
		NP	DINA	DAA
− 20	30 min	37 ± 3 (13)	1560 ± 100 (11)	253 ± 14 (11)
	1 day	60 ± 2 (22)	4460 ± 280 (32)	606 ± 28 (26)
	14 days	98 ± 4 (35)	5960 ± 300 (42)	729 ± 31 (32)
	28 days	106 ± 6 (38)	6500 ± 410 (46)	882 ± 47 (40)
5	30 min	51 ± 2 (18)	2590 ± 150 (18)	353 ± 16 (15)
	1 day	110 ± 3 (39)	8760 ± 520 (62)	895 ± 41 (39)
	14 days	168 ± 8 (60)	9860 ± 640 (70)	1590 ± 80 (69)
	28 days	192 ± 7 (69)	11000 ± 680 (78)	1690 ± 80 (74)
25	30 min	121 ± 6 (43)	6140 ± 450 (44)	1180 ± 60 (51)
	2 hr	205 ± 9 (73)	9300 ± 560 (66)	1620 ± 70 (71)
	4 hr	224 ± 8 (80)	10900 ± 600 (77)	1740 ± 80 (76)
	16 hr	240 ± 10 (86)	11300 ± 600 (80)	1810 ± 90 (79)
60	10 min	161 ± 7 (57)	9400 ± 51 (67)	1380 ± 70 (60)
	30 min	188 ± 8 (67)	10700 ± 600 (76)	1580 ± 80 (69)
	60 min	201 ± 11 (72)	11300 ± 600 (80)	1700 ± 80 (74)
	2 hr	231 ± 14 (83)	11400 ± 700 (81)	1740 ± 90 (76)
95	5 min	162 ± 7 (58)	9960 ± 480 (71)	1490 ± 70 (65)
	10 min	184 ± 9 (66)	10500 ± 600 (74)	1660 ± 90 (72)
	30 min	221 ± 13 (79)	12000 ± 700 (85)	1790 ± 100 (78)
	60 min	226 ± 12 (81)	10700 ± 700 (83)	1790 ± 80 (77)

Stretch film (film 1, 18 cm^2^) covered 16.5 g (18 ml) of rapeseed oil in a Petri dish and was maintained under the test conditions.

Each value is the mean ± SD of three or more determinations.

( ) is migration ratio (%) = migration level (μg/dm^2^)/material content (μg/dm^2^) × 100.

The migration levels of DINA were extremely high between 1560 μg/dm^2^ (at −20°C after 30 min) and 12,000 μg/dm^2^ (at 95°C after 30 min) and their ratios were from 11% to 85%. Under most conditions, the rapeseed oil after contact contained over 6 mg/dm^2^ of DINA.

The migration levels of DAA were between 253 μg/dm^2^ (at −20°C after 30 min) and 1810 μg/dm^2^ (at 25°C after 16 hr) and their ratios were from 11% to 79%. The migration tendency of DAA was similar to that of DINA.

The migration levels of NP were from 37 μg/dm^2^ (at −20°C after 30 min) to 240 μg/dm^2^ (at 25°C after 16 hr), and their ratios were from 13% to 86%. More than 50% of NP in the film was released into the oil at 5°C after 14 days, 25°C after 2 hr, 60°C after 10 min, and 95°C after 5 min. Even at −20°C, 35% of the NP was released into the oil after 14 days. The migration ratios of NP into rapeseed oil were similar to those of DINA and DAA under all conditions.

The migration levels increased with test temperature and time. However, DINA and DAA levels decreased at 95°C, they were presumed to have vaporized at this temperature.

### 3.4 Migrations of NP, DINA, and DAA from PVC stretch film into various foods during cold storage

Foods were covered with PVC stretch film and stored at 5°C for 24 hr. This condition evaluated foods stored in a refrigerator at a supermarket and at home until consumption the next day. Their migration levels and ratios are shown in Table 4.

**Table 4:** Migrations of NP, DINA, and DAA from polyvinyl chloride stretch film into foods in refrigerator

Food type	Food	Fat content(%)	Migration level (µg/dm^2^) (Migration ratio, %)		
			NP	DINA	DAA
Vegetable & fruit	Japanese radish	—	8 ± 0 (2.9)	23 ± 3 (0.1)	2 ± 0 (0.1)
	Pumpkin	—	18 ± 4 (6.4)	26 ± 3 (0.2)	6 ± 1 (0.3)
	Pineapple	—	10 ± 1 (3.6)	27 ± 7 (0.2)	5 ± 2 (0.2)
	Melon	—	17 ± 1 (6.1)	36 ± 4 (0.3)	6 ± 1 (0.3)
Cooked food	Cooked radish with fried tofu	0.5	21 ± 3 (7.6)	321 ± 33 (2.2)	36 ± 2 (1.6)
	Meat sauce	3.8	26 ± 12 (9.3)	467 ± 75 (3.3)	102 ± 34 (4.4)
	Hamburger	13.6	35 ± 8 (13)	1010 ± 148 (7.2)	160 ± 16 (7.0)
	Potato salad	13.7	44 ± 10 (16)	956 ± 100 (6.8)	184 ± 23 (8.0)
	Croquette	15.6	44 ± 2 (16)	1430 ± 90 (10)	168 ± 16 (7.3)
Meat & fish	Minced chicken white meat	1.3	43 ± 6 (15)	185 ± 32 (1.3)	54 ± 4 (2.3)
	Minced tuna	7.6	93 ± 9 (33)	5190 ± 370 (37)	563 ± 42 (24)
	Minced pork	12.5	67 ± 8 (24)	2980 ± 230 (21)	345 ± 29 (15)
Mean			35	1050	136

Stretch film (film 1, 18 cm^2^) covered food (20 g) in a Petri dish at 5°C for 24 hr.

Each value is the mean ± SD of three or more determinations.

( ) is migration ratio (%) = migration level (μg/dm^2^)/material content (μg/dm^2^) × 100.

The migration levels of DINA were 23–36 μg/dm^2^ (migration ratio, 0.1%–0.3%) in vegetables and fruits, 321–1,430 μg/dm^2^ (2.2%–10%) in cooked food, and 185–5190 μg/dm^2^ (1.3%–37%) in meat and fish. DINA migrated at high rates into minced tuna and minced pork. The migration levels of DAA were 2–6, 36–184 and 54–563 μg/dm^2^, respectively, and their migration ratio were 0.1%–0.3%, 1.6%–8.0% and 2.3%–24%, respectively. The highest migration levels of DAA were also into minced tuna. The migration rates of DINA and DAA from PVC stretch film demonstrated similar tendencies.

In contrast, the migration levels of NP were 8–17 μg/dm^2^ (2.9%–6.1%) into vegetables and fruits, 21–44 μg/dm^2^ (7.6%–16%) into cooked food, and 43–93 μg/dm^2^ (15%–33%) into meat and fish. The difference among the various foods was within only 12‐fold. On the other hand, the migration levels of DINA were 23–36 μg/dm^2^ (0.1%–0.3%) into vegetables and fruits, 321–1430 μg/dm^2^ (2.2%–10%) into cooked food, and 185–5190 μg/dm^2^ (1.3%–37%) into meat and fish, and the migration levels of DAA were 2–6 μg/dm^2^ (0.1%–0.3%), 36–184 μg/dm^2^ (1.6%–8.0%), and 54–563 μg/dm^2^ (2.3%–24%), respectively. The difference among the various foods was 370‐fold of DINA and 240‐fold of DAA.

NP migrates at a much higher ratio into non‐fatty food than DINA and DAA because of the hydrophilicity of its phenol group. The octanol/water partition coefficient (log Kow) and water solubility of NP are 3.88–4.77 and 6.2 mg/L, respectively (USFDA 2005), although those of DINA are 9.24 and under 1 mg/L (Maag et al., 2010).

Overall, minced tuna demonstrated the highest migration levels of NP, DINA, and DAA, although its fatty content was only 7.6%. This content was lower than that of hamburger (13.6%), potato salad (13.7%), croquette (15.6%), and minced pork (12.5%). The migration levels demonstrated a slight correlation with fatty contents, however, it was speculated that other factors, such as the surface condition of foods and fat properties, would also contribute.

The migration levels of NP, DINA, and DAA into vegetables and fruits were much higher than those into water and even higher than those into 20% ethanol. These observations revealed that water is not an adequate simulant for general food such as non‐acidic and non‐fatty food and suggested that 20% ethanol would be more appropriate.

### 3.5 Migrations of NP, DINA, and DAA covering hot foods and heated in a microwave oven

Migration levels of NP, DINA, and DAA in hot foods were determined after films were used to immediately cover cooked hot foods, such as radish with fried tofu, meat sauce, hamburger, and croquette, and then left for 30 min at room temperature (Table 5). The migration levels of NP were 26–37 μg/dm^2^ (9.2%–13%), those of DINA were 617–1130 μg/dm^2^ (4.4%–8.0%), and for DAA were 55–139 μg/dm^2^ (2.4%–6.0%).

**Table 5:** Migrations of NP, DINA, and DAA from polyvinyl chloride stretch film into hot food

Foods	Migration level (μg/dm^2^) (Migration ratio, %)		
	NP	DINA	DAA
Cooked radish with fried tofu	27 ± 3 (9.6)	617 ± 94 (4.4)	55 ± 7 (2.4)
Meat sauce	26 ± 6 (9.2)	873 ± 116 (6.2)	115 ± 18 (5.0)
Hamburger	27 ± 5 (9.6)	1100 ± 160 (7.8)	135 ± 21 (5.9)
Croquette	37 ± 11 (13)	1130 ± 92 (8.0)	139 ± 20 (6.0)

Stretch film (film 1, 18 cm^2^) covered just‐cooked hot food (20 g) in a Petri dish, stored at room temperature for 30 min.

Each value is the mean ± SD of three or more determinations.

( ) is migration ratio (%) = migration level (μg/dm^2^)/material content (μg/dm^2^) × 100.

The migration levels into foods that were heated in a microwave oven at 100 V, 600W (ER‐M3, Toshiba Co., Japan) for 20–60 s just after boiling were determined (Table 6). The migration levels of DINA were 889–1550 μg/dm^2^ (6.3%–11%), those of DAA were 140–232 μg/dm^2^ (6.0%–10%), and those of NP were 24–48 μg/dm^2^ (8.5%–17%).

**Table 6:** Migrations of NP, DINA, and DAA from polyvinyl chloride stretch film into food during microwave heating

Foods	Heatingtime (s)	Migration level (μg/dm^2^) (Migration ratio, %)		
		NP	DINA	DAA
Cooked radish with fried tofu	20	48 ± 8 (17)	1270 ± 130 (9.0)	224 ± 33 (9.8)
Meat sauce	30	26 ± 3 (9.3)	889 ± 189 (6.3)	140 ± 22 (6.0)
Hamburger	60	24 ± 2 (8.5)	1330 ± 280 (9.5)	180 ± 52 (7.8)
Croquette	60	46 ± 10 (16)	1550 ± 368 (11)	232 ± 96 (10)

Stretch film (film 1, 18 cm^2^) covered cold food (20 g) in a Petri dish, heated in a microwave oven for 20−60 s just after boiling.

Each value is the mean ± SD of three or more determinations.

( ) is migration ratio (%) = migration level (μg/dm^2^)/material content (μg/dm^2^) × 100.

The migration levels into each food under these two conditions were almost the same levels as those after storage at 5°C for 24 hr. In the usual use of films for storing or cooking, NP, DINA, and DAA migrated approximately under 17% of the film contents and NP migrated more than DINP and DAA. However, they would transfer much higher amounts into foods that would be heated longer as shown in Table 2.

### 3.6 Contents of NP and DINA in food

In the late 1990s to 2000, NP and DINA were determined in various types of food in Japan. The NP levels were reported to be 0.07–0.723 mg/kg (Sasaki et al., 1999) and the DINA levels were reported to be <0.005–20.2 mg/kg (Saito et al. 2002). Meanwhile, their levels of migration from PVC stretch films into food were 8–93 μg/dm^2^ for NP and 23–5190 μg/dm^2^ for DINA at 5°C for 24 hr. If it is assumed that 1 kg of food would be in contact with a 6 dm^2^ film, the level of contaminants in food would be 0.048–0.558 mg/kg for NP and 0.14–31.1 mg/kg for DINA. In this manner, the contents of NP and DINA in food and the migration levels of NP and DINA from PVC films were approximately consistent. In other words, it could be estimated that most of NP and DINA in Japanese food is derived from PVC wrapping films on that time.

### 3.7 The daily intakes of NP and DINA from PVC stretch films

The daily intakes of NP and DINA from these PVC stretch films around the year 2000 were estimated on the basis of these results and a survey of the types of food packaging used and the prevalence on Japanese market (Hirose et al., 2008). A typical Japanese individual (50 kg body weight) consumes approximately 2 kg of food per day. In the survey, 2.3% of food (46 g) was packed with PVC film, although the ratio of surface area/food weight of PVC film is much greater than those of other types of packaging, and its use is unevenly distributed (e.g., most fresh food was wrapped with PVC films in supermarkets). Therefore, we assumed it on the 3.5 times outside to 1/6 kg (167 g). Because US and EU regulations assume that 1 kg of food would be covered with 6 dm^2^ of plastic packaging, 1/6 kg of food would be covered with 1 dm^2^ of PVC film. The estimated daily intakes for the Japanese were 35 μg/person/day (0.70 μg/kg bw/day) for NP and 1050 μg/person/day (21 μg/kg bw/day) for DINA. The tolerable daily intake (TDI) of NP has not been established yet; however, the Danish Institute of Safety and Toxicology has proposed 5 μg/kg bw/day (Maag et al. 2010). TDI of DINA has also not been established, but a TDI 300 μg/kg bw/day has been established for a similar chemical, di‐2‐ethylhexyl adipate (DEHA) in EU (SCF 2000). Incidentally, the estimated daily intake of NP and DINA should not be associated with any safety concerns.

## Conclusion

Most PVC stretch films in the Japanese market during the 1980s and 1990s contained approximately 100–400 mg/g of DINA as a plasticizer and 1–2.6 mg/g of NP as a degradation product of tris(nonylphenyl) phosphite (TNP). The samples used in this study were typical films of this time. In 2002, the Japan Vinyl Goods Manufacturers Association decided to discontinue the addition of TNP to PVC stretch films. Since then, NP has not been detected in PVC stretch films in Japan, although a high content of DINA has continued.

The migration levels from PVC stretch films into foods were 23–5,190 μg/dm^2^ for DINA, 2–563 μg/dm^2^ for DAA, and 8–93 μg/dm^2^ for NP at 5°C for 24 hr. They migrated at high levels into lipid‐soluble simulants, rapeseed oil, and fatty foods owing to their lipophilic property. Furthermore, plasticizers migrated only in very low amounts into aqueous simulants and non‐fatty food. However, NP migrated much more easily into them owing to its phenolic function.

Their migration levels increased with the test temperature and time; however, even in the refrigerator at 5°C for 24 hr, 39%–62% of the film content migrated into rapeseed oil and 24%–37% were found in minced tuna. The migration levels in hot foods or foods that were heated in a microwave oven were not so high (2.4‐17%), which were almost similar levels to those after storing at 5°C for 24 hr.

The estimated daily intakes from this study were 35 μg/person/day (0.70 μg/kg bw/day) of NP and 1050 μg/person/day (21 μg/kg bw/day) of DINA. In comparison to proposed TDI of NP 5 μg/kg bw/day by the Danish Institute of Safety and Toxicology, and TDI of DEHA, chemical similar to DINA, 300 μg/kg bw/day in EU, the estimated daily intake of NP and DINA should not be associated with any safety concerns.

## Conflict of Interest

None declared.
